# *Educational Resource Review:* MSD’s The Steward—Episode 3—AMS in Northern Ireland

**DOI:** 10.1093/jacamr/dlab064

**Published:** 2021-05-17

**Figure:**
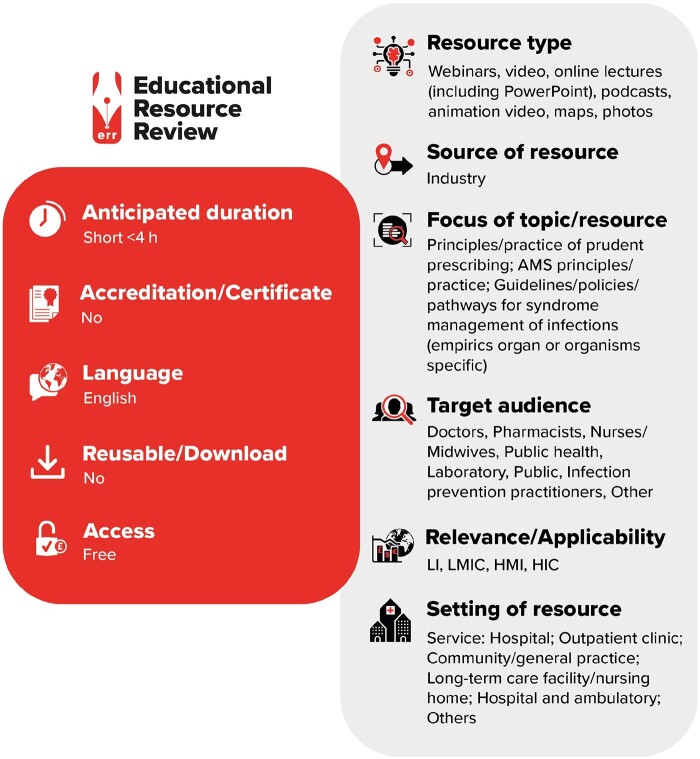


LI, low-income countries; LMIC, low- and middle-income countries; HMI, high- and middle-income countries; HIC, high-income countries.

**Resource web link:** https://www.youtube.com/watch?v=1dyW1bviUa8 (Full classification scheme available at: http://bsac.org.uk/wp-content/uploads/2019/03/Educational-resource-review-classification-scheme.pdf)

**WHO region and country (World Bank):** European Region, UK (HIC)

## Peer review commentary

MSD’s ‘The Steward’ is a series of interview style podcasts that explores topics relating to infection prevention and control (IPC) and antimicrobial stewardship (AMS). Episode 3, AMS in Northern Ireland, introduces a Clinical Pharmacist as the special guest to discuss the challenges and successes observed within antimicrobial stewardship programmes in Northern Ireland. The 45 min podcast uses language that makes this resource universally inclusive and provides clarification on acronyms used for listeners outwith healthcare.

The podcast is more informative and awareness raising than educational in content and provides geographical context to AMS. The unique challenges faced in combating antimicrobial resistance in Northern Ireland are well described, as is the impact COVID-19 has had in this area. A recurring theme within the resource is the importance of engaging a multi-professional workforce to influence and deliver a successful AMS programme.

As the resource is hosted on a video platform, it could be enhanced by providing visual content of some of the tools, projects and papers described within the narrative to further engage the user.

Overall, this is an informative resource which describes the past, present and future AMS activities in Northern Ireland. When accessed alongside the other episodes in the series, the listener will gain a broad understanding of the AMS and IPC position within the UK.

